# Critical Feminist Service-Learning: A Physical Activity Program in a Woman’s Prison

**DOI:** 10.3390/ijerph18147501

**Published:** 2021-07-14

**Authors:** Ingrid Hinojosa-Alcalde, Susanna Soler

**Affiliations:** Institut Nacional d’Educació Física de Catalunya (INEFC), Universitat de Barcelona (UB), 08038 Barcelona, Spain; ihinojosa@gencat.cat

**Keywords:** service learning, gender, social justice, higher education

## Abstract

In recent decades, increasing emphasis has been placed on the role of universities in ensuring inclusive and equitable quality education. Higher education strategies such as critical feminist service-learning (CFSL) can help achieve true university-community engagement to achieve social good while allowing students to develop academic competencies, values, and social responsibility. This study analyzes the impact of a university physical activity–based CFSL project implemented at a woman’s prison. The study combined quantitative and qualitative research methods. Eighty-one students (24 women and 57 men) participated in the project over the 3 years in which it was run (2017–2018, 2018–2019, and 2019-2020). Aspects related to the four principles of CFSL were analyzed using data collected from questionnaires and reflective learning journals completed by the students and a semi-structured interview with the prison sports instructor. Our findings show that participating in the project brought about significant changes in students’ beliefs and preconceptions about prisons, and helped them to develop their critical awareness. They also show that the project strengthened the collaborative relationship between the establishments and agents involved. In conclusion, CFSL is a powerful higher education strategy that can be used to show future educators and practitioners how sport and physical activity can drive social justice and contribute to the achievement of the United Nation’s 2030 Sustainable Development Goals.

## 1. Introduction

The Bologna process has led to numerous changes in higher education systems in recent decades. According to this new European model, universities should not only provide quality education, but also incorporate and adapt to social contexts, providing solutions that respond to local social, political, and economic needs [1]. More recently, the United Nations, within its 2030 Agenda for Sustainable Development, established 17 Sustainable Development Goals (SDGs) [2]. This blueprint underlines the crucial role that universities have in supporting and committing to the achievement of SDGs through the provision of inclusive, equitable quality education and the promotion of social learning opportunities.

Universities occupy a privileged position in today’s society and will be key to the achievement of the SDGs set out in the 2030 Agenda. As part of this role, they promote active learning that places students at the center of their own learning by connecting them to their community and promoting social justice. An example of a methodology that universities can use to contribute to the achievement of the SDGs is service-learning (SL) [3]. SL enables universities to truly engage with their community to achieve social good while providing students with the opportunity to develop academic competencies, values, and social responsibility [4]. The aim of this study is twofold: (1) to analyze the perceptions and beliefs of Physical Activity and Sport Science (PASS) students regarding physical activity in prison and (2) to analyze the perceived effects of a CFSL physical activity intervention in a woman’s prison by the PASS students and the prison sport instructor.

### 1.1. Implementation of SL Projects in Physical Education Teacher Education and Sport Science Programs

Physical activity and sport provide an ideal setting for the implementation of SL projects supporting the social inclusion of vulnerable groups [5,6]. There are numerous examples of SL projects that have been implemented within Physical Education Teacher Education (PETE) and Physical Activity and Sports Sciences (PASS) programs [7]. A recent systematic review of 31 SL projects with PETE students concluded that, despite the considerable differences between program recipients, sport as a tool for social inclusion had excellent potential for promoting the development of professional, personal, and social skills among students [8]. SL also strengthens the connection between future physical activity and sport professionals and the realities and challenges of an ever-evolving, increasingly diverse, social and educational environment.

The literature contains numerous examples of sport and physical activity programs aimed at a wide variety of populations, such as children with disabilities, older people, and schoolchildren in contexts of social exclusion [7,8,9]. A number of studies have analyzed experiences with higher education physical activity and sport SL programs in prisons [10,11]. There is, however, a lack of knowledge on the implementation of SL projects involving incarcerated women.

### 1.2. Critical Feminist Service-Learning, Women Prisoners, and Physical Activity

There is a growing body of literature calling for a critical approach to SL for social justice [12,13]. One of the key aspects of critical SL is its orientation towards social change. It is expected to accentuate the skills, knowledge, and experiences required of a student not only to participate in the community but also to become a committed, active citizen [14]. Critical SL must also strive to create true community-university partnerships throughout the planning, implementation, and evaluation phases of a project, each with its respective agents [13]. For SL to reach its full potential, it is important to avoid paternalism and to direct learning and services towards a redistribution of power and the construction of authentic relationships [12].

Critical feminist SL (CFSL) combines the principles of SL and feminist pedagogy [15], which explicitly incorporates the analysis of gender inequalities into institutional and sociocultural contexts [16]. Hauver and Iverson [15] describe four main principles underpinning CFSL:

*“(1) CFSL brings explicit attention to power relationships at work in the discursive, institutional and sociocultural contexts in which service-learning takes place. (2) CFSL utilizes consciousness-raising as a mechanism through which to gain awareness and a means through which to organize, strategize, and act. (3) CFSL emphasizes activism and promotes the development of skills necessary to work toward feminist social change. (4) CFSL is undertaken in the context of reciprocal, mutual relationships that are characterized by trust, understanding, and shared responsibility over time”*

(p. 105)

It is particularly important to integrate a critical feminist perspective into SL programs designed to meet the social needs of women in prison. Women account for just 6.6% of the prison population in Catalonia [17], and according to a recent report by the Andalusian Pro Human Rights Association (APDHA), there are a number of factors that aggravate their situation in prison [18]. The report noted that women prisoners were subject to three types of discrimination: social, personal, and penitentiary. Socially, for example, they are judged for not adhering to traditional gender roles (e.g., household chores and care) assigned to them by society. Personally, they are separated from their families, with the risk of broken ties, although there are programs that support the family unit and even allow children up to the age of 3 years to live with their mothers in the prison. As examples of penitentiary discrimination, the APDHA report [18] mentioned precarious spaces (and hence living conditions), long distances from family members, grouping together of women irrespective of their criminal profile, and difficulties accessing treatment programs or certain workshop jobs.

Physical activity and sports programs are popular among male prisoners [19,20]. Uptake is lower among women, who continue to face barriers to the practice of sport in the prison environment [21,22]. Gender inequalities in sport may therefore be even higher in prison than in society [23]. Accordingly, physical activity must be promoted among women prisoners to improve their physical, mental, and social health.

Contact with external organizations helps prisoners connect to the outside world and learn about new activities and opportunities for when they are released. These encounters can also counter the effects of prisonization. They bring a breath of fresh air and with it an opportunity to see new faces, build new relationships, and capture a glimpse of reality [20]. Finally, visits to prisons can help normalize conversations about imprisonment in the community and reduce stigmatization [24,25].

### 1.3. Context of the Intervention

The first step in the CFSL project was a meeting between the university teachers and the head of the prison sports programs from the Catalan Department of Justice to discuss possibilities for collaboration. After this initial meeting, both parties explored numerous ways to connect the university’s PASS degree to prison sports programs. It was decided to introduce the CFSL project in an optional SL subject for fourth-year students, who would be responsible for designing and implementing a physical activity program at a prison. The two establishments agreed that the intervention should target women, as this met a social need identified by the Justice Department and allowed the university to incorporate a critical feminist perspective into the project. The CFSL project was implemented at the women’s prison located closest to the university. From an educational perspective, the intervention enabled us to actively incorporate a gender perspective into the students’ curriculum and to focus on the needs and interests of adult women, who are often overlooked in PASS studies due to androcentric bias [26].

The participants in the CFSL project were the PASS students, the inmates at the prison, the prison sports instructor, and the university lecturers. The students kept a reflective learning journal in which they were encouraged to reflect on their experiences and learning throughout the design, implementation, and evaluation stages of the project.

Before designing the program, the students completed a series of information-gathering exercises and activities that challenged them to think about the prison system and gender issues in sport. The goal was to strengthen their critical thinking skills and heighten their awareness of the situation of women prisoners and their relationship with physical activity and sport. The four principles of CFSL were reinforced during these activities. The students, for example, identified the different prisons in Catalonia, studied the sociodemographic profile of their populations (men and women), and collected information on the practice of physical activity. Emphasis was placed on analyzing the power relationships present in prisons and the discrimination faced by women in both prisons and sport. Preconceptions about prisoners were discussed in a climate of reflection designed to encourage students to express themselves freely and to jointly confront and dispel prejudices and misconceptions. A worker of the Department of Justice was also invited to give a lecture to the students and offer another perspective on the context of the intervention while addressing doubts and concerns raised by the students. During these activities, the students became conscious of their prejudices towards prisoners.

Several preparatory sessions were held to design the basic structure and content of the proposed physical activity program. The prison sports instructor was then contacted to analyze the specific needs of the prison and adjust the program content accordingly. These needs were also analyzed during the students’ first visit to the prison. This preparatory phase was important for incorporating a feminist perspective into the program. The feminist perspective was introduced in the content of the physical activity program taking into account several aspects. Women’s interests were considered to select the contents and activities. Models of social intervention through physical activity were implemented aiming to promote feelings of group cohesion. The forms of physical activity that were carried out were diverse, such as cooperative motor skills games, awareness-raising motor skills games and fitness health-oriented activities with musical support. Students employed a gender-inclusive language in the design and implementation of the program. The session dynamic provided pee-to-peer relationships between students and inmates seeking to break the hierarchy among the instructors and participants.

The program consisted of two weekly physical activity sessions held over 4 weeks. The sessions were carried out Mondays and Wednesdays from 9:30 am to 12 am. The interventions were run by groups of two to three students, both of whom led and participated in the sessions. This participation was considered an essential part of the program, as it favored interpersonal relationships between the students and the prisoners. Between 10 and 15 women voluntarily enrolled in the program during each academic course.

On completion of the program, the students received feedback from their academic tutors, the women who participated, and the prison sports instructor. The students’ tutor and sports officer also met to evaluate the overall intervention.

## 2. Materials and Methods

This study combined quantitative and qualitative research methods. The quantitative data were collected using a purpose-designed questionnaire, while the qualitative data were obtained from the students’ reflective learning journals and a semi-structured interview held with the prison sports instructor.

### 2.1. Participants

A total of 81 students (24 women and 57 men) from the university’s PASS degree program at Institut Nacional d’Educació Física de Catalunya (INEFC) participated in one of three editions of the CFSL program implemented in a woman’s prison between 2017 and 2020. All participants had enrolled in the optional subject “Leisure and social environment”. The prison sports instructor was interviewed in 2020.

### 2.2. Data Collection and Analysis

Prior to the CFSL program, students were asked to complete an ad hoc 13-item questionnaire on sociodemographic characteristics and perceptions and knowledge about sport in prison. The answers were analyzed in SPSS (version 24) and summarized using descriptive statistics.

All the students kept a reflective learning journal (RLJ) throughout the SL program. They were asked to record thoughts and insights about their learning experience and to include anecdotes and reflections about the training process, relationships with others, and personal impressions about their participation in the program.

A semi-structured interview(I) was held with the prison sports instructor on completion of the three editions of the CFSL intervention. The interview was tape-recorded and transcribed verbatim.

The students’ and the sports instructor’s names were removed from the data and all participants were assigned a pseudonym to protect their privacy.

Qualitative data were thematically analyzed using the six steps proposed by Sparkes and Smith [27]: (1) familiarization with the data, including transcription and reading of interviews; (2) generation of initial codes based on previous research and “discovery” of other codes through inductive analysis; (3) grouping of codes into main themes; (4) review of themes; (5) definition and naming of themes; and (6) production of report. The data were coded and analyzed in Atlas.ti 7. The most significant statements were chosen to exemplify the themes in the results section.

## 3. Results

Four themes related to the basic principles of CFSL [17] were identified: (1) knowledge (or lack of), beliefs, and preconceptions about prisons; (2) change, including the development of critical awareness among students; (3) the future, by cultivating professionals with personal and social responsibility; and (4) the synergy of university–prison collaborations.

### 3.1. Knowledge (or Lack of), Beliefs, and Preconceptions about Prisons

Most students (75.21%) had never visited a prison. The most visited prisons were Quatre Camins and La Modelo, both men’s prisons. Quatre Camins is known to students who specialize in handball, as they have the option of participating in a project involving matches between inmates and students played at the prison and the university facilities [28]. Some students were familiar with the building of La Modelo, as they had visited it during a guided tour when it had stopped housing prisoners in 2017.

When asked about the purpose of prisons in Catalonia, 61.73% of the students pointed to rehabilitation and social reintegration. Nonetheless, we also observed some myths and misconceptions: 12.35% of students thought that the purpose of prisons was to decrease inappropriate behavior, while 12.35% said it was to contain and correct, and 9.88% said it was to punish.

The vast majority of students (88.24%) thought that prisoners performed physical activity, 1.96% thought that they did not, and 9.80% did not know. Physical activity and prisons were not things that students connected naturally, it was far from their thoughts. As noted by Miguel in his reflective learning journal “I had never thought about whether prisoners did physical activity or not, or about what type they might do”.

When asked about the type of physical activity they thought prisoners participated in, the students gave various answers: team sports (mentioned by 27.85%), body-building (12.66%), and all types of activities (12.66%); 11.39% said that they did not know.

Influenced by a lack of knowledge and stereotypical portrayals of prisons in the media, the students felt uneasy before their intervention. Mentions of nervousness and fear about entering the prison and interacting with the prisoners were common.

*When we arrived, they locked the door behind us (there are two doors everywhere); I felt uneasy about this and scared because if they lock the door, it’s for a reason. I began to think that they were dangerous, that they could hurt us.*

(RLJ-Lorena)

Other students’ accounts illustrated their stereotypical views of prisons and concerns about how the prisoners might react. Alba, for example, said:

*I had never had the chance to enter a prison before, I wondered what it would be like. Would it be like in the films? Would I have to walk along corridors, past doors? The night before my intervention, my mind was spinning, I felt uneasy, insecure, and I kept asking myself how the girls would react when I proposed the activities.*

(RLJ-Alba)

As seen, the main sensations felt by the students before entering the prison and interacting with the prisoners were unease and nervousness. Even though they were aware that their emotions were due to prejudices and preconceptions, they still felt uneasy before their first direct contact.

### 3.2. Change: Development of Critical Awareness among Students

The classroom activities challenged the students to start thinking about the discrimination faced by women in prison.

*Also, I was really shocked to learn that just 6.6% of prisoners are women compared with 93.4% for men; this difference is crazy and partly explains why women are more marginalized in prisons.*

(RLJ-Octavio)

The students were starting to become conscious of the marginalization faced by women prisoners and to recognize the challenges of working with people with very different characteristics to what they were used to.

*…and we are surprised that we are going to be able to work with women prisoners, considering that the percentage of women prisoners is much lower than that of men prisoners, we are happy to be given this challenge, I’m sure it will be a very positive and motivating experience.*

(RLJ-Manuel)

The journal reflections also showed how the students were starting to empathize with the women and understand their vulnerability, particularly those with children under the age of 3.

*In addition, before entering the prison we saw children aged between 0 and 3 leaving the mothers’ module, this is where women prisoners with children live, and children up to the age of 3 go to sleep there. It must be very hard for a mother to see her child leaving for school every day and to think that you are not going to see them again until nighttime or know anything else about them. What if something happens to them? Or worse still, when they are older (>3 years) and can’t sleep there anymore and you only see them for X minutes a week.*

(RLJ-Andrea)

The classroom activities were also designed to confront individual attitudes towards prisoners, and the different debates about myths and beliefs generated changes in awareness and attitudes.

*Personally, another point made by the lecturer that affected me emotionally was when she asked us if we would choose a prisoner if we were hiring at our company. I said no without giving it a second thought. I am very impulsive about thoughts and decisions like this and I’m grateful for what the teacher said after she heard our answers […] nowadays, anyone can find themselves in their situation, and it could be one of us one day who needs help.*

(RLJ-Nuria)

After the implementation of the project, most of the students remarked on how the experience had changed their opinions on prisons and prisoners. The following testimony shows how their preconceptions about how the prisoners would react to the intervention were wrong:

*I had imagined that the prisoners weren’t going to do anything, not even listen, that they were going to be reluctant to do anything we told them, but the truth is that they listened to us all the time, it was not difficult to convey ideas to them, and they behaved well.*

(RLJ-Diego)

Many students also had stereotypical views of prisons and prisoners based on what they had seen in films or series. These views were dismantled by visiting the prison and participating in the project, as evidenced by the following comment:

*Before I went to do the session, I pictured the prison like the prisons I’d always seen on TV: inmates all wearing the same uniforms or suits, only able to move around the prison if accompanied by a guard, guards on every corner, etc. When I got there, however, I was pleasantly surprised to see that this was not the case, all the inmates were free to move around the prison, there were no guards along the corridors, and the women were able to do whatever they wanted during the permitted times.*

(RLJ-Jairo)

The students even mentioned how participating in the physical activity sessions alongside the prisoners created a positive climate that made it easy to interact.

*We were received really well, much better than I expected, and maybe that’s what surprised me most, as they were laughing and joking all the time, and when the session ended, some of them gave us a couple of kisses on the cheek and hugged us as if we’d been friends for life.*

(RLJ-Ismael)

Elsa also mentioned how her perceptions about life in prison and prisoners had changed:

*These interventions have really changed how I feel, how I view life in prison, and on the contrary, I find it hard to understand how people with whom I had a perfectly normal relationship could have made a mistake at some point in their lives that led them here.*

(RLJ-Elsa)

One of the aspects that made the greatest impression on the students was the bond they formed with the prisoners and seeing how their visits affected them.

Ana, for example, said that when they went to the prison for the second time, about a week or two after the first visit, the prisoners remembered them and were happy to see them: “We entered the prison and some of the prisoners greeted us, they remembered us!” (RLJ-Ana)

Vicente mentioned his surprise at the gratitude he felt from the prisoners:

*When I left, I was surprised that some of them came to say goodbye and hug us at the door, I liked this and saw it as a gesture of gratitude.*

(RLJ-Vicente)

The students also reflected on the range of emotions they felt on completing the project in their journals. On the one hand, they remarked on how satisfied and proud they were about having participated in this project, while on the other, they mentioned that they had become aware of how difficult it was to be deprived of one’s freedom. Enrique, for example, said:

*When our intervention was over, it was time to say goodbye. This was one of the worst and most uncomfortable moments for me. Simply because I couldn’t say “See you soon!” “See you!” “See you next time!” ... It’s situations like this that bring you back to the reality you had managed to avoid through the physical activity sessions, and you realize that they are there, that you probably won’t ever see them again, and that they are locked in.*

(RLJ-Enrique)

This appreciation illustrates how the CFSL project provided the students with the opportunity to develop their professional skills, but also strengthened their sense of personal and social responsibility, as discussed in greater detail below.

### 3.3. The Future: Cultivating Professionals with Personal and Social Responsibility

Designing and implementing the physical activity program for women prisoners allowed the students to connect and apply what they had learnt during their degree to the real world. As Antonio said: “I’ve been able to apply things I learned in other subjects on the degree and actually implement them during the sessions” (RLJ-Antonio).

The students, for example, proposed activities they believed would interest the women, even though they did not master these activities. There were several cases in which the students stepped out of their comfort zone and designed new activities adapted to the needs and interests of the prisoners. In doing this, they broke away from traditional androcentric offerings and created a program that involved women. One of the activities, for example, consisted of prisoner-led Zumba sessions, so it was actually the women who were teaching the students.

In some cases, the satisfaction of having participated in the SL project awakened an interest to continue working with the prison on a voluntary basis. As explained by Vicente:

*I could see both during and after the session that I hit it off well with them and that this feeling was mutual, they also felt at ease with me. I felt so satisfied and so at ease that I decided right there and then that I wanted to come back and that I would do anything I could to do that.*

(RLJ-Vicente)

While several students mentioned an interest in continuing to volunteer at the prison, others who had not had particularly high expectations at the start remarked on how the project had influenced their professional development. They drew attention, for example, to both the quantity and quality of what they had learned throughout the experience.

*Looking back and thinking about the bigger picture, my personal experience with this project has been very satisfying. I liked it much more than I thought I would, I enjoyed the experience and learned a lot, not so much at the theoretical level, more in terms of values. Looking ahead, career-wise, I know that this is not what I want to do, but I definitely know that what I have learned from the two sessions is unprecedented. And, equally important, things you experience for the first time always leave a mark on you.*

(RLJ-David)

Some students also reflected on the professional skills needed to develop sports programs as a social intervention in vulnerable contexts:

*As a sports professional, it is important to know how to work with different groups, to be flexible, creative, able to resolve conflicts, and communicate effectively, clearly, and warmly.*

(RLJ-Maite)

The students also remarked on how the experience had prompted them to think about their careers in physical activity and sport. Some of them mentioned a particular interest in continuing to work in sport as a tool for social inclusion and integration in vulnerable groups.

*After participating in this project, I can see myself working in areas related to social inclusion and integration. I like working to improve and help society and people who for whatever reason have not found their place in society and have had problems in their lives that may have led them to take complicated decisions.*

(RLJ-Erica)

This interest in social inclusion and integration was also reflected in an increased demand by students for work placements in this field and the completion of final degree projects on the subject.

### 3.4. Synergy: Collaboration between Prisons and University

The CFSL project has served to strengthen the reciprocal and collaborative relationships between the university and the prisons. One of the most notable effects from the perspective of the prisons is the impact that visits from the outside have on the prisoners, as they provide them with a break from routine, an opportunity to escape from the day-to-day at the center. Some of the students were aware of this impact. As Miriam explained:

*From what I noticed, and heard from sports instructor, they liked having people coming in to do the activities, it breaks their routine and allows them to disconnect for a while.*

(RLJ-Miriam)

The prison sports instructor also mentioned how important these external collaborations were and how important it was that they involved physical activity:

*Just having people come in from the outside is already an innovation. What’s more, the cooperative games they organized are done very little here, normally for a special event, these sessions were therefore new for them and were a different way of doing physical activity.*

(I-Sports instructor)

The instructor also remarked on how these new activities, which the students led and participated in, had a different effect on the prisoners.

*The difference I would like to point out is how happy the inmates were with the sessions. As I’ve already explained, the prison is very small, they see a lot of each other, and they are often in the same class and ignore each other. With your sessions, you succeeded in getting inmates who do not “get on” to share really fun moments, of opposition and collaboration. Very important values for living together. Most of the women actively participated in the experience and enjoyed it.*

(I-Sports instructor)

The alliance created between the university and the sports unit of the Catalan Department of justice as a result of the CFSL project has led to other forms of collaboration between the establishments. The prisons, for example, have arranged visits to the university to show the prisoners the campus and its facilities and the studies on offer, and technical staff and current and former prisoners have also participated in several seminars held at the university. Finally, the university provides a channel for job offers that might attract students with experience in the prison system.

## 4. Discussion

Our study has shown the impact of a CFSL program on the higher education of PASS students and on a community partner, in this case a woman’s prison.

The data collected before the launch of the program showed that the students were largely unfamiliar with the Catalan prison system and had a number of stereotypical views and preconceptions about prisoners. This lack of familiarity with the prison system among university students has been previously described [29]. Very few had visited a prison, or knew how many prisons there were in Catalonia or where they were located, even a small percentage of students had visited one of the prisons as part of another university project [28]. Our results show that prisons are still taboo and, on occasions, invisible. As indicated by Garcia-Tardon et al. [30], young people continue to see prisons in a negative light and have very little knowledge or information about this subject. A majority of the students recognized that the purpose of prisons in Catalonia was rehabilitation and reintegration, although a third mentioned other objectives more closely linked to punishment. The students knew that the prisoners did sport and physical activity, but did not know what type. They were also unaware of the existence of a framework program. This lack of knowledge with prison reintegration programs among university students has been reported in the literature [24,29].

The students’ view of prisons and prisoners were shaped by social stereotypes and in many cases by what they had seen in the cinema or on the news. Their early journal entries illustrate their prejudices towards female prisoners, supporting previous reports by Fernandez et al. [10]. They also highlight the emotional impact of these preconceptions, such as the fear and nervousness they felt before implementing the program.

In relation to critical awareness, our findings also show how the CFSL project led to significant changes in the students’ perceptions of prisons and prisoners. As evidenced in the literature [4,6,10,31], SL projects help break down prejudices about populations at risk of social exclusion and increase awareness of the power relationships that exist. To achieve this deconstruction and consciousness, it is particularly important to draw attention to the complex power relationships and gender inequality issues that exist in prisons [12,14]. Similarly to Aramburuzabala et al. [4], we found that creating opportunities for reflection helps students take stock of gender inequalities to understand the privileges that they themselves enjoy.

Overall, as demonstrated by several studies of CFSL projects applied to populations at risk of social exclusion [4,31], participation in a program like the one described in this study favors the cultivation of professionals with a sense of personal and social responsibility. It also favors the acquisition of social and civic skills [7]. Emotional impact is an important contributor to the development of these skills in the penitentiary context, as it helps people understand themselves and others, and to express values and grow personally [11].

Participation in the project also enhanced professional development, as it gave students the opportunity to apply their learnings to a real-life situation and to contemplate, for the first time perhaps, a career where they could use physical activity and sport to promote social inclusion among vulnerable groups. Previous studies have linked the development of professional skills to participation in SL projects, as this allow students to test themselves in the real world [3,8,32].

The students in our case came to understand that incorporating a gender perspective into the physical activity sessions they were preparing for the woman’s prison would help improve participation and commitment. Numerous authors have stressed the importance of incorporating a gender perspective into the design of prisons and prison activities [16,21,22]. Enhancing participation in physical activity programs at women’s prisons is important, as sport and physical activity can improve living conditions [20,21], favor social development [33], and provide an escape from the routine of prison life [19,34].

Overall, the data collected over the years show that projects of this type have transformative potential on two fronts: on the one hand, they engage students in social change, while on the other, they contribute to personal and professional growth. In line with Boyle-Baise’s proposals [14], the students not only designed and implemented a physical activity program for a woman’s prison, but also acquired a new perspective on prisoners and learned how they could use sport and physical activity to contribute to social justice [3,15].

Finally, and in line with the principles of CFSL [14,15,16], our experience shows that SL interventions have even wider repercussions: they generate reciprocal relationships between institutions and community partners, strengthening trust, understanding, and shared responsibility.

## 5. Conclusions

Sport-based CFSL interventions implemented in a woman’s prison can generate powerful experiences for all agents involved: students, prisoners, sports instructors, justice departments, and universities. Nevertheless, there were some factors that disrupted the performance of the CFSL program. First, the background of most of the students was more related to traditional sport activities and children. Thus, the students had difficulties adjusting the activities of the program to the needs and interests of women. Second, the participation of women inmates in the physical activity intervention was irregular due to the characteristics of their daily lives in prison (e.g., family visits, justice issues). Third, the bureaucracy and permissions required to enter into the prison are slow and difficult, and do not allow changes during the implementation.

In terms of the continuity of the program, students who take part in the CFSL project are more likely to focus their final degree projects or their practicums in relation to sport, social inclusion, and gender equality. It is also important to highlight the role of the voluntary service that some students decide to continue with after implementing the program, which enhances their experience.

In conclusion, CFSL interventions are a forceful form of civic engagement and empowerment that enable universities to actively contribute to the achievement of several of the 2020 Agenda SDGS, namely, Quality Education (SDG 4), Gender Equality (SDG 5), and Reducing Inequalities (SDG 6). They favor social change and activism and help equip future educators and practitioners with the skills needed to accelerate progress towards the 2030 Agenda for Sustainable Development through physical education and sport.

## Data Availability

The data presented in this study is not available to preserve the participants privacy.
